# Partial Weight-Bearing in Female Rats: Proof of Concept in a Martian-Gravity Analog

**DOI:** 10.3389/fphys.2020.00302

**Published:** 2020-04-03

**Authors:** Carson Semple, Daniela Riveros, Janice A. Nagy, Seward B. Rutkove, Marie Mortreux

**Affiliations:** ^1^Harvard Medical School – Beth Israel Deaconess Medical Center, Department of Neurology, Boston, MA, United States

**Keywords:** partial weight-bearing, ground-based studies, spaceflight, rats, females, muscle

## Abstract

Many studies have investigated the physiological response to microgravity in both astronauts and animals. However, while space agencies have sought to deploy more women on their missions; animal models rarely include females studies or comparisons between males and females. Therefore, we exposed adult female rats to 2 weeks of partial weight-bearing at either 100% of their normal loading (PWB100) or 40% of their normal loading (PWB40), corresponding to Martian gravity-analog, and assess muscle function, force and histomorphometry. Females exposed to PWB showed an 11.62% decline in hindlimb grip force associated with an 11.84% decrease in soleus myofiber size after 14 days of exposure, while maintaining normal blood oxygenation and stress levels. This pilot study represents the first experiment designed to understand the muscular disuse associated with a partial reduction in mechanical loading in female rats, and the first step needed to develop successful mitigating strategies.

**New and Noteworthy:**

This research article describes the first use of quadrupedal partial weight-bearing in female rats. This study demonstrates the feasibility of partial gravity analogs in females and allows for future investigations about the impact of sex on muscle deconditioning due to reduced mechanical loading.

## Introduction

Despite NASA’s being created in 1958, and the first American man, Alan Shepherd, having entered space in 1961, the first woman to serve as an astronaut, Sally Ride, did not reach space until 1983 (Muller, 2005). NASA is currently trying to bridge this gap, and recently realized its first all-female spacewalk aboard the international space station (ISS) (Zraick, 2019), with plans to send the first woman to the Moon by 2024 (NASA, 2017).

Similarly, ground-based models, which are crucial in order to better understand the muscle deconditioning that occurs in space, have had a strong male bias, and rare studies have used hindlimb unloading in adult females (Peres-Ueno et al., 2017). Our recently developed rat partial weight-bearing (PWB) analog has proven to be a reliable (Mortreux et al., 2018) and consistent (Mortreux et al., 2019a) model, and when combined with the hindlimb suspension via pelvic harness (HLS), can be used to represent an additional period in microgravity (Mortreux et al., 2019c), mimicking a trip to and from these extraterrestrial bodies. However, this PWB model has only been used in adult male rats thus far (Mortreux et al., 2019b).

It is well known that gender differences are an important factor to consider when investigating physiological alterations. Indeed, sex hormones are known to play an important role in skeletal muscle homeostasis, thus creating differences in both muscle function and muscle histomorphology (Anderson et al., 2017). Indeed, testosterone has a strong anabolic role in the skeletal muscle (Herbst and Bhasin, 2004), while estradiol has been shown to inhibit muscle protein synthesis (Tipton, 2001). In Wistar rats, it has been shown that males have myofibers with greater cross-sectional area than females (Deschenes et al., 2018), and that they display a greater proportion of type 1 myofibers (Simard et al., 1987; Nakano et al., 1995; Novák et al., 2010), especially in the soleus, one of the muscles that atrophies most rapidly in response to mechanical unloading. Since unloading-induced atrophy primarily affects oxidative (type 1) myofibers (Rosa-Caldwell and Greene, 2019), and leads to a fiber switch from type 1 to type 2 (Mortreux et al., 2019b), it is likely that partial mechanical unloading will yield different results in females compared to male rats, and thus demand for specific mitigating strategies.

Here, we investigated the physiological alterations in adult female Wistar rats after 2 weeks of exposure to PWB at either 40 or 100% of normal loading, and quantified muscle function, force, and morphology. This level has been chosen based on the high expertise obtained in our laboratory, and because it models Martian mechanical loading, a target for space agencies in the near future. Additionally, we assessed the effects of PWB on food intake, body weight, and parameters associated to the activation of the hypothalamic-pituitary-adrenal (HPA) axis. Finally, we used electrical impedance myography (EIM) to assess muscle health both *in vivo* and *in vitro* and report here the first findings in a rat model of PWB.

## Materials and Methods

### Animals

All experimental protocols were approved by the Beth Israel Deaconess Medical Center Institutional Animal Care and Use Committee.

Fourteen Wistar female rats (Charles River Laboratories, Wilmington, MA, United States) weighing 282.96 ± 5.43 g (14 week of age) were obtained and housed in a temperature-controlled facility (22 ± 2°C) under a 12:12 h light-dark cycle (lights on starting at 7:00 am) with water and regular chow provided *ad libitum.* Rats were allowed to acclimate to their single housing for 48 h before being exposed to different levels of PWB for 14 days according to our previously described protocol (Mortreux et al., 2018). On day 0 (baseline), each rat was placed into one of two conditions, to ensure equal distribution of the body weights across the groups: normal loading with full harness (PWB100) and 40% of normal loading (PWB40). Achieved PWB level was calculated daily by recording the fully loaded weight and the unloaded weight on a digital animal scale as previously described (Mortreux et al., 2019a), and the chain length was adjusted in case of a deviation greater than 5% of the desired PWB level. All experiments were performed during the light-phase.

All experiments requiring anesthesia were performed using inhaled isoflurane (1.5–3.5%) + oxygen; body temperature was controlled by a water therapy pad (Gaymar Orchard park, NY, United States) set at a constant temperature of 37°C (Fisher Scientific, Hampton, NH, United States). After 14 days of PWB, animals were euthanized by CO_2_ inhalation according to IACUC guidelines.

### Grip Force Measurements

Rats were positioned with their front or rear paws on a 50 N capacity digital grip force meter (Chatillon, Largo, FL, United States) and gently pulled backward until they released their grip. The bar was attached to a force transducer, measuring the peak force generated. Three tests were performed with a 30-s period between each trial to avoid muscle fatigue and the peak force from each trial was averaged.

### Foot Plate Dorsal Force Generation

Under anesthesia, rats were placed on a force place (dual Mode Muscle Lever System, Aura Scientific, Aurora, ON, Canada), and their left foot was taped onto a foot plate. Using monopolar electrodes (28G, Natus Medical Incorporated, Pleasanton, CA, United States), a tetanic supramaxial stimulation of the peroneal nerve at a frequency of 200 Hz for 200 ms was applied and the maximal torque was recorded. These measurements were obtained weekly and the area under curve (AUC) was calculated and compared to the pre-suspension (baseline) values.

### Calf Circumference

Under anesthesia, animals were placed in a prone position with the left hindlimb taped at a 45° angle. Fur overlaying the leg was shaved using a hair clipper (Braintree Scientific, Braintree, MA, United States), and the calf circumference was measured three times using a suture thread at the tibial mid-shaft. The length of each thread was measured with a micrometer (Fisher Scientific, Hampton, NH, United States) and the average was recorded.

### Serological Measurements

Blood was collected from the tail vein in awake animals between 8:45 and 9:15 am. Blood glucose was determined (Contour Blood Glucose Meter, Ascensia Diabetes Care US, Inc., Parsippany, NJ, United States) and blood was collected using a heparin-coated capillary (Fisherbrand, Fisher Scientific, Hampton NH, United States). Blood samples were centrifuged for 5 min at 8000 rpm, plasma was collected and stored at −20°C upon further analysis. Plasma corticosterone levels were assessed using ELISA kits (Crystal Chem #80554, Elk Grove Village, IL, United States).

### Electrical Impedance Myography

*In vivo* and *ex vivo* EIM measurements in the left gastrocnemius were made using the mView System (Myolex, Inc., Boston, MA, United States), and obtained at 41 frequencies ranging from 1 kHz to 10 MHz. For *in vivo* measurement, rats were anesthetized and a needle array, adapted from a mouse needle array described elsewhere (Kapur et al., 2018) (1 mm spacing, 2 mm depth, with 1 mm of the tips left exposed) was inserted in the left gastrocnemius either in a longitudinal or transverse direction relative to the myofibers orientation. Two measurements were acquired in each direction and averaged. Impedance parameters (phase, resistance and reactance) were assessed at 50 kHz. For *ex vivo* measurement, a 1 cm^3^ piece of fresh gastrocnemius was placed into a dielectric cell, adapted from the mouse cell described elsewhere (Sanchez et al., 2014). The tissue fibers were oriented parallel to the flow of current for longitudinal measures and perpendicular to the flow of current for transverse measures.

### Tissue Collection

Immediately after euthanasia, organs were harvested and weighed on a precision analytical balance (Fisher Scientific, Pittsburgh, PA, United States) and placed in 10% formalin for 48 h at 4°C before being transferred to PBS for further histological analysis.

### Muscle Histomorphometry

The left soleus, gastrocnemius, tibialis anterior and triceps brachii muscles were embedded in paraffin and immunohistochemical analysis was performed on full cross sections obtained from the muscle belly using anti-collagen VI (ab6588, Abcam, Cambridge, MA, United States) and anti-slow skeletal myosin heavy chain (ab11083, Abcam, Cambridge, MA, United States). Images were acquired at 20× using an epifluorescence microscope (Zeiss Axio Imager M1), and were analyzed with FIJI (ImageJ, NIH) to determine the myofibers cross-sectional area (CSA) using the muscle morphometry plug-in (Anthony Sinadinos using Eclipse IDE), with the experimenter blinded for partial-weight bearing level.

### Statistical Analysis

The number of animals for this pilot study was decided after performing a power analysis based on our data obtained in age and strain-matched males. Indeed, to detect a significant change in grip force, our power analysis revealed that *n* = 5 per group would give us 80% power, and that *n* = 7 per group would yield 90% power. Based on muscle mass (soleus), our analysis showed that *n* = 5 per group would yield a 90% power. All longitudinal measures were analyzed using 2-way repeated measures ANOVAs followed by Sidak’s *post hoc* tests unless specified otherwise. Terminal outcomes were analyzed using two-tailed *t*-tests, once they passed the normality Shapiro-Wilk’s test. All results are presented as mean ± SEM unless otherwise specified and were analyzed using GraphPad Prism 8.1. (GraphPad Software, La Jolla, CA, United States) and considered significant when *p* < 0.05.

## Results

**FIGURE 1:**
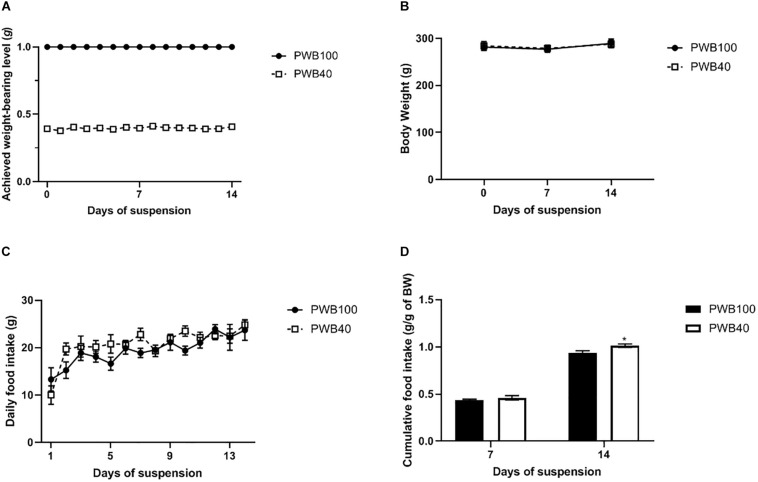
Weekly evolution of **(A)** achieved weight-bearing level, **(B)** body weight, **(C)** daily food intake, and **(D)** weekly cumulative food intake per gram of body weight, in animals exposed to PWB100 and PWB40. Results are presented as mean ± SEM and were analyzed using 2-way RM-ANOVAs followed by Sidak’s *post hoc* test. *p < 0.05 vs PWB100, *n* = 7 per group.

Throughout the 14-day experiment, the achieved weight-bearing level was steady (Figure 1A) with a coefficient of variation of 2.10% for the PWB40 group. PWB40 animals did not experience any significant variations in their body weight, both compared to the control group and to their pre-suspension value (Figure 1B); similarly, daily food intake was similar across both groups (Figure 1C). Interestingly, while cumulative food intake at 7 and 14 days did not differ between the two groups, when normalized to the animals’ body weight, it was significantly greater in the PWB40 group at 14 days (Figure 1D).

### Muscular System

**FIGURE 2:**
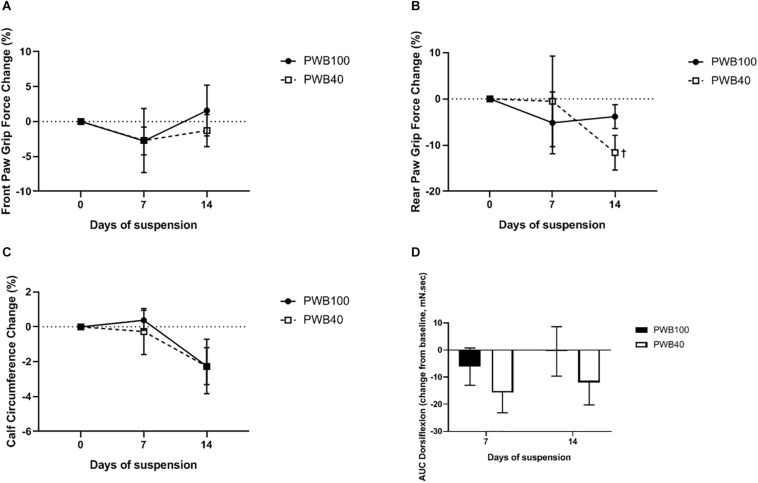
Weekly evolution of muscle function including **(A)** front paw grip force, **(B)** rear paw grip force, **(C)** calf circumference, and **(D)** area under curve (AUC) of the maximal dorsiflexion recorded after a tetanic stimulation, in animals exposed to PWB100 and PWB40. Results are presented as mean ± SEM and were analyzed using 2-way RM-ANOVAs followed by Sidak’s *post hoc* test (A, C, D, *n* = 7 per group) or with a mixed effects model followed by Sidak’s *post hoc* test. ^†^*p* < 0.05 vs baseline, *n* = 6–7 per group **(B)**.

Weekly, longitudinal analysis of muscle function was performed using grip force measurements in the fore- (Figure 2A) and hind- limbs (Figure 2B), calf circumference measurements (Figure 2C) and tetanic foot dorsiflexion (Figure 2D). Over the course of the experiment, we failed to detect a significant change in front paw grip force or calf circumference, both between groups and compared to their baseline. Rear paw grip fo rce remained stable in animals exposed to PWB100; however, after 14 days, the PWB40 group displayed a significant decrease compared to their baseline, but not to the control group (−11.62 ± 3.76%, *p* < 0.05). Finally, recording of dorsiflexion torque in response to tetanic nerve stimulation did not yield significant results despite a clear trend toward a loss of force in the group exposed to PWB40. Indeed, PWB40 led to a reduction of the AUC of 15.65 ± 7.47 and 11.90 ± 8.33 mN.sec after 7 and 14 days, respectively, while the control group only displayed a reduction of 6.08 ± 6.88 mN.sec after 7 days, and of 0.49 ± 9.12 mN.sec after 14 days of PWB100.

**FIGURE 3:**
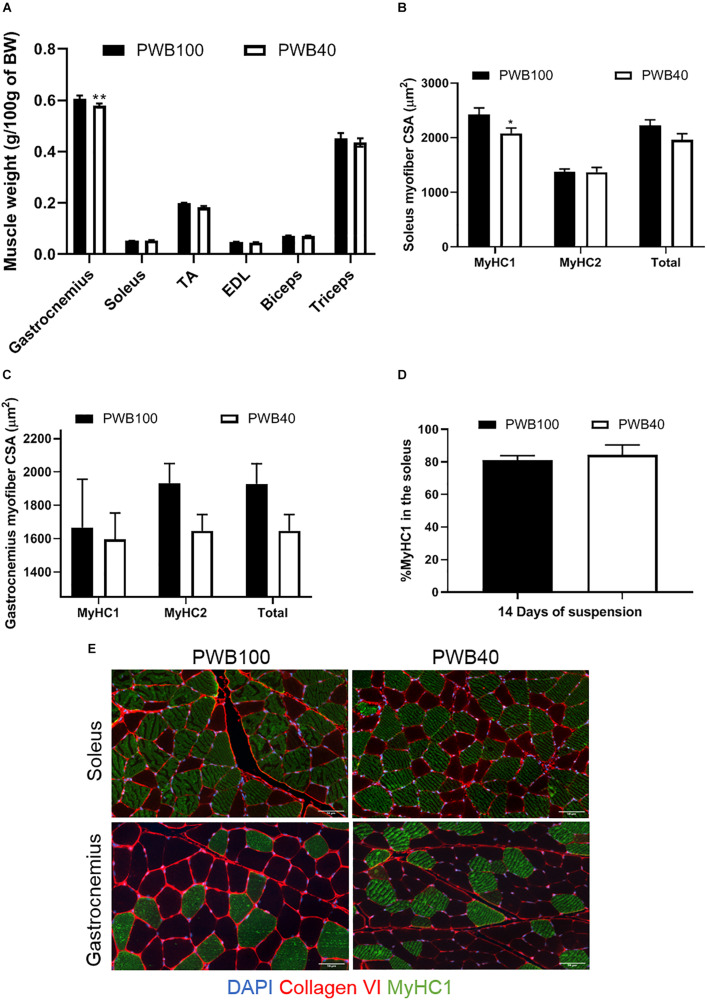
Terminal assessment of muscle weight and myofiber size. Normalized muscle weight after 14 days of exposure to PWB100 or PWB40 in the front and hind limbs **(A)**. Myofiber CSA in the gastrocnemius **(B)** and soleus **(C)** muscles, percentage of type-1 fibers in the soleus **(D)** and representative micrographs of the immunohistochemistry staining performed, and **(E)**. Results are presented as mean ± SEM and were analyzed using unpaired Student’s *t*-test. *, **p < 0.05, and *p* < 0.01 vs PWB100, respectively, *n* = 7 per group.

At the end of the experiments, fore- and hind- limbs muscles (including the gastrocnemius, soleus, tibialis anterior (TA), extensor digitorum longus (EDL), biceps brachii and triceps brachii) were dissected, weighed, and normalized to the animals’ body weight (Figure 3A). Amongst the hindlimb muscles, only the gastrocnemius displayed a significant atrophy after 2 weeks of exposure to a reduced mechanical load. Soleus weight did not vary, while TA and EDL weights were reduced in the PWB40 group but failed to reach significance. Using immunohistochemistry, we characterized the gastrocnemius (Figure 3B) and soleus (Figure 3C) myofibers using histomorphometry and were able to detect a significant reduction of the soleus myofiber CSA that solely impacted slow-twitch (type I) fibers, which led to an overall diminution of 11.84% of the CSA across all myofibers, without impacting the percentage of type I fibers overall (Figure 3D). In the gastrocnemius, we observed a clear but insignificant reduction of the CSA of the fast-twitch (Type II) fibers, which led to an overall diminution of 14.62% of the mean CSA across this muscle. Representative micrographs used for the histomorphometric analyses are displayed in Figure 3E.

### Physiological Parameters of Stress

Several physiological parameters associated with the activation of the HPA axis were assessed weekly in our animals (Table 1). Neither plasma parameters (i.e., blood glucose and corticosterone) nor heart rate varied significantly between groups or over the course of the experiment. However, while blood glucose remained extremely stable, our results highlight that plasma corticosterone levels displayed a greater variability.

**TABLE 1:** Weekly measurement of plasma parameters, foot oxygenation, and heart rate; and wet mass of the adrenal glands and spleen after 14 days of PWB.

*Days*	0	7	14
**Blood glucose (mg/dL)**			
PWB100	108 ± 3	105 ± 6	108 ± 8
PWB40	110 ± 4	103 ± 9	112 ± 7
**Plasma corticosterone (ng/mL)**			
PWB100	57.46 ± 11.02	58.44 ± 21.27	48.01 ± 15.90
PWB40	82.41 ± 14.10	37.11 ± 7.51	34.87 ± 11.01
**Heart rate (bpm)**			
PWB100	398 ± 17	398 ± 10	409 ± 11
PWB40	397 ± 28	409 ± 7	425 ± 11
**Foot SpO2 (%)**			
PWB100	98.0 ± 0.7	98.6 ± 0.2	98.7 ± 0.2
PWB40	98.8 ± 0.1	97.8 ± 0.8	98.7 ± 0.2
**Adrenal glands wet mass (g/100g of BW)**			
PWB100			0.043 ± 0.002
PWB40			0.047 ± 0.001
**Spleen wet mass (g/100g of BW)**			
PWB100			0.289 ± 0.019
PWB40			0.273 ± 0.013

*n = 7 per group. Results are presented as mean ± SEM and were analyzed using 2-way repeated measures ANOVAs followed by Sidak’s test (longitudinal measures) or unpaired Student’s t-test (terminal measures). Significant results were not detected.*

Finally, wet mass of the adrenal glands and spleen were obtained after 14 days of exposure to PWB, as these organs usually exhibit hypertrophy in response to stress. Similar to our longitudinal observations in male rats (Mortreux et al., 2020), we were not able to detect a significant change between our two experimental groups.

### Electrophysiological Analysis

Intra-muscular EIM was used weekly to assess muscle health *in vivo* through examination of the 50 kHz values (Table 2). Control rats (PWB100) displayed stable values for phase, resistance, and reactance in both the longitudinal and transverse directions over the course of the study. However, animals exposed to PWB40 displayed a relative but not significant increase in longitudinal resistance after 7 days of unloading, and a significantly lower resistance after 14 days. None of the transverse parameters were significantly different.

**TABLE 2:** EIM values at 50 kHz in both longitudinal and transverse directions.

*Days*	0	7	14 *in vivo*	14 *ex vivo*
**Longitudinal phase**				
PWB100	20.1 ± 0.8	20.1 ± 0.8	20.1 ± 0.5	21.9 ± 1.4
PWB40	18.9 ± 0.8	20.5 ± 0.5	19.8 ± 0.5	17.1 ± 1.1*
**Longitudinal resistance**				
PWB100	142.1 ± 3.2	144.2 ± 2.2	140.0 ± 1.5	193.7 ± 7.1
PWB40	138.9 ± 3.1	145.8 ± 2.1	136.8 ± 1.4^‡^	194.8 ± 9.3
**Longitudinal reactance**				
PWB100	52.3 ± 3.2	52.9 ± 2.8	51.3 ± 1.6	77.5 ± 5.5
PWB40	47.9 ± 2.8	54.6 ± 2.2	49.4 ± 1.5^‡^	60.2 ± 5.2*
**Transverse phase**				
PWB100	15.7 ± 0.6	16.5 ± 0.6	17.0 ± 0.5	35.6 ± 1.7
PWB40	16.6 ± 0.7	17.2 ± 0.7	16.3 ± 0.7	35.1 ± 1.0
**Transverse resistance**				
PWB100	115.0 ± 4.7	117.3 ± 2.6	114.4 ± 2.4	316.8 ± 33.5
PWB40	111.1 ± 6.4	110.6 ± 2.9	108.8 ± 2.0	395.3 ± 32.8
**Transverse reactance**				
PWB100	32.5 ± 2.2	34.8 ± 1.7	35.0 ± 1.5	232.7 ± 32.1
PWB40	33.5 ± 3.1	34.5 ± 2.2	31.8 ± 1.4	282.0 ± 30.3

Results are presented as mean ± SEM and were analyzed using 2-way RM-ANOVAs followed by Sidak’s *post hoc* test. ^‡^*p* < 0.05 vs day 7, *n* = 7 per group. *Ex vivo* EIM values at 50 kHz in both longitudinal and transverse directions obtained from the freshly excised gastrocnemius muscle. Results are presented as mean ± SEM and were analyzed using unpaired Student’s *t*-test. *: *p* < 0.05 vs PWB100, *n* = 7 per group. *n* = 7 per group. Results are presented as mean ± SEM and analyzed using 2-way repeated measures ANOVAs followed by Sidak’s post hoc test. *p* < 0.05 vs day 7 (in vivo), and with unpaired Student’s *t*-test *p < 0.05 vs PWB100 (ex vivo).

During *ex vivo* analysis, we observed a significant decrease in longitudinal phase and reactance in animals exposed to PWB40 compared to the fully loaded controls (PWB100). However, longitudinal resistance, as well as the transverse values, were not affected by 14 days of exposure to partial mechanical unloading.

## Discussion

In this pilot study, we demonstrate the first use of a PWB model for adult female rats, at one level of partial unloading (40%) for 14 consecutive days compared to fully loaded animals. Similarly to our previous observations of PWB in male rats (Mortreux et al., 2018), we showed that the PWB system is extremely steady and reliable over time with a coefficient of variation (CoV) of 2.1% during the 2-week experiment. Despite being greater than the 1.2% CoV value in male animals previously reported by our group for periods of 2 to 4 weeks (Mortreux et al., 2019a), the CoV reported here remains well below our target range of ±5%.

Overall, females appear to tolerate well the period of partial mechanical unloading. While PWB led to a transient body weight loss in male rats (Mortreux et al., 2018, 2019a,b,c), we did not observe any significant fluctuations of the females’ body weight, either compared to the control group or to their pre-suspension baseline. This sex-based difference has been reported previously after exposure to 14 and 30 days of hindlimb unloading (Il’ina-Kakueva, 2002; David et al., 2006; Chang et al., 2018; Deschenes et al., 2018), and in our study, was further associated with a similar food intake, which was eventually greater at the end of the experiment in the PWB40 group when normalized to animals’ body weight, compared to the PWB100 controls.

Like other physiological systems (Hefferan et al., 2003; Glenmark et al., 2004; Foley et al., 2005), muscle displays sex-based differences in both phenotype and mechanism during states of enforced disuse (Ploutz-Snyder et al., 2014; Rosa-Caldwell and Greene, 2019). Since few studies have aimed at investigating male and female rats concomitantly, and because of the various models and durations employed, a consensus has yet to be reached regarding the effect of sex differences in muscle deconditioning. Here, we show that exposure to PWB40 does not significantly impair muscle function compared to the controls, as characterized by voluntary grip force in both the fore- and hind- limbs. However, reduced mechanical loading leads to a significant loss of rear paw grip force after 2 weeks. These results, while preliminary, are rather different than what was observed in our previous studies with males, where we highlighted that hindlimb grip force decreases by over 20% after 14 days of exposure to PWB40 (Mortreux et al., 2018, 2019c). Previous research has shown that females’ muscles are more resistant to fatigue compared to their male counterparts (Glenmark et al., 2004) and hindlimb unloading studies conducted on Wistar rats of both sexes also emphasized the differences in bone adaptation, where 14 days of hindlimb unloading led to a significant reduction in femoral bone mineral density in male, but not in female Wistar rats (David et al., 2006). Finally, human studies highlighted the fact that the time-course of muscle deconditioning during unloading could be impacted by sex (Ploutz-Snyder et al., 2014). Interestingly, our terminal assessments of several muscles revealed that muscle and myofiber size seemed differently impacted in female rats compared to those observed in male rats in the same conditions. In the hindlimbs, the postural soleus muscle is usually the first impacted by total and mechanical unloading (Fitts et al., 2000, 2001; Mortreux et al., 2019a), where it is associated with a preferential decrease of type-I myofiber size (Ploutz-Snyder et al., 2014; Baehr et al., 2017) and a typical myofiber switch from type I (slow) to type II (fast) fibers (McDonald et al., 1994; Desplanches, 1997; Shenkman, 2016; Mortreux et al., 2019b).

In our pilot study, females exposed to 14 days of PWB40 did not exhibit a decreased soleus mass; however, we were able to detect a significant atrophy of the type I myofibers, which was not associated with a myofiber type switch throughout the muscle (Figure 3D). The absence of fiber-type switch has previously been described in female rats (Il’ina-Kakueva, 2002; Deschenes et al., 2018), though others have not identified this (Chang et al., 2018). Taken together, our analysis of the soleus muscle allows us to hypothesize that in females, preferential myofiber atrophy occurs prior to myofiber-type switch and gross muscle atrophy, however, it remains to be confirmed in longer experiments. Muscle histomorphometry further identified a non-significant reduction of 14.83% in type 2 fibers and an overall reduction in myofiber CSA of 14.62% in the gastrocnemius, along with a significantly decreased muscle mass.

In addition to the differences in the soleus response to decreased mechanical loading, our study did not establish the presence of a significant atrophy of the biceps and triceps brachii after 14 days of exposure. While most of our previous work focused on the effects of PWB on the hindlimb muscles function and strength, we recently demonstrated that varied degrees of mechanical unloading sustained for 28 days lead to a dose-dependent decrease of the myofibers’ CSA in the triceps brachii (Mortreux et al., 2020). In an effort to establish if this atrophy was detectable after 14 days of exposure to PWB40 in females, we did shift our efforts from the quadriceps femoris to the forelimbs muscles (Mortreux et al., 2019a). Future studies should investigate if females display a rapid atrophy of the quadriceps femoris wet mass and if longer exposure induces significant changes in forelimb muscles mass.

EIM is finding increasing value for the assessment of muscle health in humans (Tarulli et al., 2005; Rutkove et al., 2017; Shefner et al., 2018). In a few seconds, localized impedance measurements can be obtained and include resistance (*R*) and reactance (*X*). While resistance is associated with the effect of fluids (both intra and extra cellular) on the applied current, reactance is a parameter dependent on the cell membranes, which act as capacitors. The phase (*P*) is calculated based on these two values according to the equation: *P* = arctan *X*/*R* (Nie et al., 2006). Often, EIM will be measured both longitudinally and transversally compared to the myofibers’ orientation, thus providing information about muscle anisotropy (Esper et al., 2006). It has previously been shown to be sensitive to disuse in rats (Li et al., 2013) and has been used by our laboratory to detect similar changes in the muscles of mice onboard the space shuttle and exposed to hindlimb unloading (Sung et al., 2013). Here, we evaluated whether EIM could be used as a longitudinal biomarker for changes in muscle health in female rats exposed to PWB. While we identified differences in two EIM features in the muscle *ex vivo* at 14 days, we did not observe significant differences *in vivo*, although a trend toward increasing resistance was found at 1 week, and such alteration has been found to be associated to muscle disuse and atrophy (Tarulli et al., 2009). In previous *in vivo* work in rats studying change using a tail-suspended hindlimb model, we used surface electrode arrays. Conversely, for the first time, we used a needle array to avoid skin irritation relating to the repeated use of depilatory agent. Further refinement of this *in vivo* needle based approach may be required; nevertheless, the observed changes, although not significant, may be meaningful, especially based on the remarkable stability of the values in the PWB100 group.

When we first reported the development and characterization of the rat PWB model, we did not investigate the stress level of the male animals; however, we subsequently performed this analysis (Mortreux et al., 2020). These experiments demonstrated that, regardless of the partial unloading level, PWB carried out with the use of a pelvic harness in lieu of the traditional tail suspension, did not elicit a chronic stress response nor impact hindlimb blood flow. Here in females, we observed similar results based on heart rate, hindlimb blood oxygenation, plasma levels of glucose and corticosterone, and spleen and adrenal glands mass.

Taken together, we demonstrate for the first time the feasibility of conducting PWB studies in adult female rats, and the impact of PWB40 on hindlimb muscle health. Indeed, we are able to report that the PWB model is well tolerated by adult females, and offers a steady, reliable, and durable reduction in mechanical loading. While we were expecting to observe sex-based differences compared to what has been previously described in strain and age-matched males, our pilot study offered some answers whilst having some important limitations. First, our analog model only mimics the decrease mechanical load on the animals, and does not expose them to a lower gravity (e.g., 0.4 g); it is also not associated with other parameters that would be involved in a Martian mission such as the exposure to galactic cosmic rays (GCR). Moreover, as in males, and similarly to what is observed in space-flown rodents, this model does not elicit a cephalic fluid shift, which is an important component of human physiology in space. However, we were able to detect a significant alteration of the hindlimbs muscle strength, size, and morphometry in response to PWB, although our sample was limited and, despite a power analysis, did not allow us to detect significant changes in all parameters.

The study reported here represents the first step in determining the longitudinal time-course of the physiological adaptations to periods of reduced mechanical loading, and should be followed by an extensive characterization of the physiological response of females to reduced mechanical loading. Moreover, we did not cycle our animals nor did we assess if their reproductive cycles were impacted in response to reduced mechanical loading. In the future, using both males and females concomitantly in PWB paradigms will help us understand the sex-based differences in physiological alterations during extra-terrestrial missions and design optimized countermeasures ensuring the safety of all astronauts during their journeys in space.

## Data Availability Statement

All datasets generated for this study are included in the article/Supplementary Material.

## Ethics Statement

The animal study was reviewed and approved by Beth Israel Deaconess Medical Center Institutional Animal Care and Use Committee.

## Author Contributions

MM, DR, and CS performed the experiments. MM, CS, and JN analyzed the results. MM designed the methodology, prepared the figures and wrote the manuscript. MM, JN, and SR revised the manuscript. SR obtained the funding. All authors were involved in the revision of the manuscript and approved its final form.

## Conflict of Interest

SR has equity in, and serves a consultant and scientific advisor to Myolex, Inc., a company that designs impedance devices for clinical and research use; he is also a member of the company’s Board of Directors. The company also has an option to license patented impedance technology of which SR is named as an inventor. The authors have no other relevant affiliations or financial involvement with any organization or entity with a financial interest in or financial conflict with the subject matter or materials discussed in the manuscript apart from those disclosed.
