# Corrigendum: Partial Weight-Bearing in Female Rats: Proof of Concept in a Martian-Gravity Analog

**DOI:** 10.3389/fphys.2020.00672

**Published:** 2020-07-03

**Authors:** Carson Semple, Daniela Riveros, Janice A. Nagy, Seward B. Rutkove, Marie Mortreux

**Affiliations:** Harvard Medical School – Beth Israel Deaconess Medical Center, Department of Neurology, Boston, MA, United States

**Keywords:** partial weight-bearing, ground-based studies, spaceflight, rats, females, muscle

In the original article, there was a mistake in Table 1 as published. The value displayed for heart rate on day 0 for the PWB40 group was incorrect. The incorrect value was 349 ± 59, it has been replaced by the correct value 397 ± 28. The corrected Table 1 appears below.

**Table 1:** Weekly measurement of plasma parameters, foot oxygenation, and heart rate; and wet mass of the adrenal glands and spleen after 14 days of PWB.

**Days**	**0**	**7**	**14**
**Blood glucose (mg/dL)**			
PWB100	108 ± 3	105 ± 6	108 ± 8
PWB40	110 ± 4	103 ± 9	112 ± 7
**Plasma corticosterone (ng/mL)**			
PWB100	57.46 ± 11.02	58.44 ± 21.27	48.01 ± 15.90
PWB40	82.41 ± 14.10	37.11 ± 7.51	34.87 ± 11.01
**Heart rate (bpm)**			
PWB100	398 ± 17	398 ± 10	409 ± 11
PWB40	397 ± 28	409 ± 7	425 ± 11
**Foot SpO2 (%)**			
PWB100	98.0 ± 0.7	98.6 ± 0.2	98.7 ± 0.2
PWB40	98.8 ± 0.1	97.8 ± 0.8	98.7 ± 0.2
**Adrenal glands wet mass (g/100g of BW)**			
PWB100			0.043 ± 0.002
PWB40			0.047 ± 0.001
**Spleen wet mass (g/100g of BW)**			
PWB100			0.289 ± 0.019
PWB40			0.273 ± 0.013

*n = 7 per group. Results are presented as mean ± SEM and were analyzed using 2-way repeated measures ANOVAs followed by Sidak's test (longitudinal measures) or unpaired Student's t-test (terminal measures). Significant results were not detected*.

The authors apologize for this error and state that this does not change the scientific conclusions of the article in any way. The original article has been updated.

